# Comparative Analyses Between the Smoking Habit Frequency and the Nucleolar Organizer Region Associated Proteins in Exfoliative Cytology of Smokers' Normal Buccal Mucosa

**DOI:** 10.1186/1617-9625-2-4

**Published:** 2004-03-15

**Authors:** Renata Pittella Cançado, Liliane Soares Yurgel, Manoel Sant'Anna Filho

**Affiliations:** 1 Department of Oral and Maxillofacial Surgery, School of Dentistry, Pontificia Universidade Católica do rio Grande do Sul, Brazil; 2 Department of Estomatology, School of Dentistry, Pontificia Universidade Católica do rio Grande do Sul, Brazil; 3 Department of Oral Pathology, School of Dentistry, Pontificia Universidade Católica do rio Grande do Sul, Brazil; 4 Av. Rio Branco, 984/802. Vitória, ES. Bracil. CEP: 29050-642

## Abstract

An evaluation of the cellular alterations in the smoker's oral mucosal cells was performed. Exfoliative Citology technique were applied and the cytologic smears stained with silver for quantitative analyses of Argyrophilic nucleolar organizer regions. (AgNORs). Cytologic smears were collected from two anatomic sites, mouth floor and tongue border with the purpose of relating the frequency of smoking with the quantitative analyses of the AgNORs. This study showed that the average number of AgNORs/nucleus is related with the number of cigarettes per day in the mouth floor of smoker's. These results suggest a possible relation between the number of cigarettes per day and an increase rate of cellular proliferation in the oral mucosal cells.

## Introduction

Cancer has been considered a Public Health problem all over the world, as this disease affects at least 9 million people and kills about 5 million every year, being currently the second cause of death in most of the countries (not considering the external causes of death) [1]. The incidence of cancer tends to raise about 100% within the next 20 years, if prevention and control measures are not taken [2]. The efforts made towards cancer prevention are based on monitoring the changes in exposure to risk factors related to the tumor development, being traditionally done by health education, on an individual or group basis, through media or other sources [3,4]. In spite of these efforts and the fact that the mouth is an easily accessed region for examination, diagnosis of oral cancer is still obsolete and about half of the diagnosed patients die from it. [5,6].

Exfoliative cytology is a diagnosis complementary exam, based on the observation of cells that are constantly exfoliating from the epithelium, as a consequence of this tissue's natural maturation process [3,7]. The dental literature of the 1960s and 1970s contained numerous reports on the use of oral cytology as a diagnostic approach. However, low sensitivity and specificity precluded the general adoption of microscopic cytology for the detection of primary or recurrent oral cancer [8-14].

According to Girod [15], if genetic changes are accumulated during carcinogenesis and precede the malignant transformation, markers of these molecular or cellular changes should be identified in specific stages of the disease. Spaffort, Koch, Califano and colleagues [16] concur that molecular markers displaying instability have proven to be highly sensitive, and able to detect a single tumor cell out of 200 normal cells in the urine.

Clonal genetic alterations such as tumor suppressor, gene mutations, or microsatellite instability are a hallmark of human cancer [17]. Many studies have suggested the use of exfoliative cytology associated with molecular markers in an oral cancer prevention program, as an auxiliary resource for early diagnosis of these lesions [18,9,16,24]. Nucleolar organizer regions (NORs) are ribosomic DNA loops and associated proteins that run on the fibrillary centers and the dense fibrillary components of the cell nucleolus, during the interphase, and are responsible for the ribosomic RNA copy. The silver soaking method was developed by Howell [25], with the purpose of staining the NORs in a differentiated way. Schawarzacher [22], In 1982, Ploton [26] adapted the silver soaking technique to observe the AgNORs into a one-step technique, which allowed them to observe these structures under the optical microscope, and in 1983 concluded that the silver stained proteins were basically formed by RNA polymerase I, C23 e B23, directly involved in nucleolus DNA and ribosomic RNA copy. The use of the AgNORs silver soaking technique in exfoliative cytology finds support in the studies done by Sujathan [27], Metze and Lorand-Metze [10], Cardillo [28] and Sampaio [6].

Sciubba [29] undertook a study to evaluate the sensitivity and specificity of OralCDx (OralScan Laboratories Inc.), a computer-assisted method of analysis of the oral brush biopsy, in the detection of precancerous and cancerous lesions of the oral mucosa. The study group conducted a multicenter double-blind study comparing results of OralCDx analysis with those of scalpel biopsy of suspicious oral lesions, as well as using OralCDx on oral lesions that appeared benign clinically. The specificity for the OralCDx "positive" result was 100 percent, while the specificity for the OralCDx "atypical" results was 92.9 percent. The authors propose that this multicenter trial demonstrates that OralCDx is a highly accurate method of detecting oral precancerous and cancerous lesions. Sampaio [6] used this technique in exfoliative cytology smears collected from the clinically healthy oral mucosal cells, comparing smokers and non-smokers. These authors showed that the number of AgNORs, as well as the frequency of nuclei with more than five AgNORs is higher within the smokers group, indicating an increase in the proliferative activity of healthy oral mucosal cells in smokers. However, additional studies are needed to evaluate the importance of these findings in the oral cancer development.

Cançado, Sant'anna and Yurgel [30] showed a statistical difference in the average number of AgNORs per nucleus and the average percentage of nuclei with more than three AgNORs between smokers and non-smokers. It was not possible to correlate the smoking frequency to AgNORs variations in this study. This study, which aims at contributing to the search for methods of early diagnosis of oral cancer, proposes to evaluate through the count of AgNORs, the use of the AgNORs silver soaking technique on clinically healthy oral mucosal cell smears, obtained through exfoliative cytology technique, in patients over 50 years old, filtered cigarette smokers, and correlate the smoking habit frequency to this amount of AgNORs per nucleus. This study also might facilitate monitoring, diagnosis and consequent life improvement of these patients. The following purposes were set: (1) to correlate the smoking habit frequency in years to the amount of AgNORs per nucleus in two anatomic sites, the tounge border and the floor of the mouth; and (2) to correlate the number of cigarettes per day to the amount of AgNORs per nucleus in two anatomic sites, the tounge border and the floor of the mouth.

## Materials and methods

As previously mentioned, this study was based on collected data from previous research performed by Cançado, Sant'anna and Yurgel [30] where a detailed description of the method can be found. The sample for this study comprises individuals: (1) between 50 and 70 years old; (2) free from any systemic diseases; (3) non-alcoholic; and (4) with no early or present history of benign or malignant neoplasias. This study comprises only smokers, with smokers defined as individuals who smoke more than 20 cigarettes/day [7] or have smoked 10 cigarettes/day over 10 years. An elaborate physical intra-mouth examination was performed, and people with any sort of mucosal alterations were excluded. The group comprised 60 smokers (20 females and 40 males). Collection of smears was performed through exfoliative technique, from two anatomic sites: tongue border and mouth floor. Two slides obtained from each patient were stained using the silver soaking technique, adapted by Ploton [31] with an incubation period of 20 minutes at 45°C.

The silver soaked smears were horizontally analyzed in all of their extension, from left to right. The nuclei from the first 100 cells observed were included in the counting. Cells with no nucleus were not included. The parameters used on the counting were defined by Crocker [32], that is, the black dots inside the well-defined nuclei were counted, with the black aggregates (overlapped or merged black dots) counted as one structure. The first 20 slides were counted in a nonconsecutive way three times, to calibrate the observer. Observed for each group in each anatomic site were: (1) the average number of AgNORs per nucleus; and (2) the average percentage of nuclei with more than 3 AgNORs. The agreement rate among the observers related to the silver soaked smears reading for the AgNOR's counting, was first calculated through the Friedman's non-parametric test. The average number of AgNORs obtained per slide and the average percentage of nuclei with more than three AgNORs were compared for each anatomic site (tongue border and mouth floor) through the Student t test (bi-caudal) for independent samples. Number of cigarettes/day and time (years) of smoking of this sample were correlated to the results found by the analysis of AgNORs, through Linear multiple regression.

## Results

This sample comprised 40 male individuals (66.66%) and 20 female individuals (33.33%). Of these 60 individuals, 43 (71.66%) were between 50 and 60 years old and 17 (28.33%) were between 61 and 70 years old. The total average of cigarettes smoked per day was 15,08 ± 4,6 cigarettes/day. The smoking habit frequency in years varied between 10 and 50 years, with an average of 27,40 ± 9,90 years. The number of AgNORs per nucleus varied between 2.39 and 1.72 AgNOR/nucleus with an average of 1.94 ± 0.13 AgNORs/nucleus in the smears collected from the tongue border and an average of 2.07 ± 0.19 AgNORs/nucleus in those collected from the mouth floor. A significant variation in the average of AgNORs/nucleus was observed among the smokers, depending on the anatomic site investigated. A higher average was found in the AgNORs/nucleus in the mouth floor (p = 0.0001). The percentage of nucleus with more than three AgNORs varied in the smoking group from 14 to 53%. The number of AgNORs per nucleus observed were calculated by the average percentage of nuclei with more than three AgNORs; this was 25.16 ± 6.01% on the tongue border, while on the mouth floor an average of 30.51 ± 8.25% was found. The results from the tongue border smears compared to the ones from the mouth floor showed a significant difference on the average percentage of nuclei with more than three AgNORs found in these two anatomic sites (p = 0.0001), the average being higher on the floor of the mouth smears. The number of cigarettes smoked per day and the smoking habit time showed no significant difference when correlated to the average of AgNORs per nucleus or the percentage of nuclei with more than three AgNORs found in this sample on the tongue border. However, on the mouth floor, the number of cigarettes smoked a day showed a significant difference (p ≥ 0,05) when correlated to the average of AgNORs per nucleus or the percentage of nuclei with more than three AgNORs. That was not true for the smoking habit time where the differences with the AgNORs number were not significant.

## Discussion

There is a need for studies that point to the direction to be adopted in oral cancer prevention and control measures and to the development of strategies that increase the population participation [33]. Monitoring the appearance of lesions in specific groups that may be classified as risk groups is an effective method of preventing cancer [10,34]. Exfoliative cytology is an adjunct method of monitoring these risk groups, in the long-term control of appearing of oral mucosal epithelium alterations when associated with molecular markers. The effectiveness of the use of quantitative techniques and markers in cytology, like DNA analysis, cytokeratin expression analysis and the use of markers like PCNA and Ki67, has been related by many authors, including Bongers [2], Ogden [20], Ogden [35] and Rosin [36]. In this study the silver soaking technique was used for an evaluation of AgNORs in smears from patients who smoked, since this is an effective method to determine ploidy and evaluate the epithelium cells' proliferative activity, as related by Hernandez-Verdum [37], Kacerovská [19], Ploton [38], Cabrini [39] and Van Diest [33]. Sujathan [27] suggested that using smears, the study of AgNORs is more precise, since the entire nucleolus can be analyzed, and not only part of it as occurs in histological analyzed tissue cuts. This affirmative is also supported by Crocker [40] and Sampaio [6].

From the affirmatives made about the usefulness of the counting of the AgNORs to establish the ploidy cellular and the proliferative standard of the epithelium [39,41,37,25,43,21,33] and based in the evaluation criteria suggested by Giri [41] and Mourad [43], we observed the variation in the number of AgNORs per nucleus in the smokers (2.39 to 1.72 AgNORs/nucleus). We also observed in this study definition of these cells as diploids and the variation of the percentage of nuclei with more than five AgNORs between 0% and 6% suggests a proliferative pattern of the epithelium that varies in intensity, but remains within a benignity pattern. Consistent differences found in the present study and in that carried out by Sampaio [6] lie in the characteristics of the sample used. In their study, Sampaio [6] examined 40 individuals divided into two groups of 20 people, smoking and non-smoking. The present study comprised 60 smoking individuals. The larger number of the sample tends to minimize the over-valuation of extreme variations in the number of AgNORs/nucleus, approaching the data obtained in the real average. Another characteristic of the sample that distinguishes the two studies is the quantity and duration of the smoking habit. In the study performed by Sampaio [6] all the individuals that comprised the sample had been smoking more than 20 cigarettes a day for over 15 years. In our study the smoking individuals that comprised the sample smoked between 10 and 20 cigarettes a day for a period that varied between 10 and 50 years. Zimmermann and Zimmermann [44], Kapczinski [45] and Ogden and colleagues [7] acknowledge the presence of cell alterations related to the number of cigarettes smoked a day and Silva [23] mentions the number of cigarettes smoked as a variable to be considered. The studies mentioned were obtained through the analysis of smears stained by Papanicolaou's Method. The results obtained in the present study showed a significant correlation between the number of cigarettes smoked a day when the smears of the mouth floor in smoking individuals are analyzed. That was not true for the tongue border smears. The study performed by Cançado, Sant'anna and Yurgel [30] indicated that a larger number of AgNORs per nucleus was observed among the smoking individuals in the mouth floor smears (2,07 ± 0,19) when compared to those collected from the tongue border (1,94 ± 0,13), with a significant difference (p = 0,0001). These findings are according to the earlier mentioned results, and corroborate the suggestion that the less keratinized oral cavity regions are more susceptible to the smoking action.

The results obtained in this study suggest that the oral mucosa is susceptible to the cigarette smoking action, a susceptibility that is reflected by a cell proliferative activity increase demonstrated by an increase on the AgNORs number per nucleus related to the number of cigarettes smoked a day in the mouth floor. The connection between the AgNORs evaluation made, dysplasias and malignant transformations remains unknown [39]. An interpretation of highly structured cell populations in AgNORs counting (cells with high turnover as the healthy oral epithelium) may be a more complex process when comparing tissues of more stable cell populations, the alteration process in a carcinoma dysplasia being more complex than just a ploidy and cell proliferation alteration [46]. The meaning of the increase in oral cavity healthy tissue cell proliferation is also questioned by Sampaio [6]. The present study suggests that there is an increase in the oral mucosal cell proliferation in smokers. More studies will be necessary to define the meaning of this increase in malignant neoplasias and epithelium dysplasias development. The present study suggests that there is an increase in the oral mucosal cell proliferation in smokers, meaning that there could be a positive relation of intensity, not periodicity, in the injuries caused by smoke in keratinized tissues.

## Conclusion

It is possible to conclude, according to the methodology used for this study and based on the results obtained, that: (1) It was not possible to correlate the smoking frequency in years to the amount of AgNORs variation in this sample in two anatomic sites, the tongue border and the mouth floor; and, the number of cigarettes per day had a significant correlation to the amount of AgNORs per nucleus in the mouth floor (p ≤ 0,05). That was not true for the tongue border smears.

## Competing interests

The authors declare that they have no competing interests.

**Figure 1:**
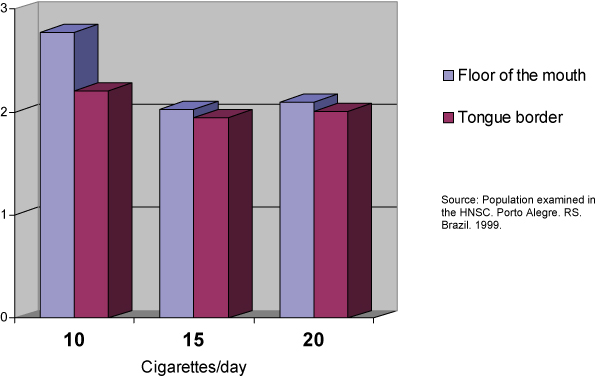
**Relationship of the number of cigarettes per day to the amount of AgNORs per nucleus by anatomic sites**. Source: Population examined in the HNSC. Porto Alegre. RS. Brazil. 1999.

**Figure 2:**
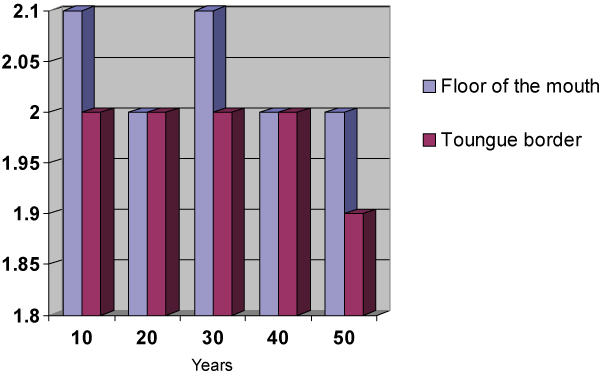
**Relationship of the smoking habit frequency in years to the amount of AgNORs per nucleus by anatomic sites**.
